# TAT for Enzyme/Protein Delivery to Restore or Destroy Cell Activity in Human Diseases

**DOI:** 10.3390/life11090924

**Published:** 2021-09-06

**Authors:** Michal Lichtenstein, Samar Zabit, Noa Hauser, Sarah Farouz, Orly Melloul, Joud Hirbawi, Haya Lorberboum-Galski

**Affiliations:** Department of Biochemistry and Molecular Biology, Faculty of Medicine, Institute for Medical Research Israel-Canada (IMRIC), The Hebrew University of Jerusalem, Jerusalem 9190501, Israel; michallic@ekmd.huji.ac.il (M.L.); samar.zabit@mail.huji.ac.il (S.Z.); noa.orbach@mail.huji.ac.il (N.H.); sarah.slama@mail.huji.ac.il (S.F.); orly.elbaz@mail.huji.ac.il (O.M.); joud.hirbawi@mail.huji.ac.il (J.H.)

**Keywords:** CPPs, TAT, TAT-MTS-MitoProtein fusion proteins, TAT-ApoProtein fusion proteins, MTS, mitochondrial diseases, apoptosis, cancer

## Abstract

Much effort has been dedicated in the recent decades to find novel protein/enzyme-based therapies for human diseases, the major challenge of such therapies being the intracellular delivery and reaching sub-cellular organelles. One promising approach is the use of cell-penetrating peptides (CPPs) for delivering enzymes/proteins into cells. In this review, we describe the potential therapeutic usages of CPPs (mainly trans-activator of transcription protein, TAT) in enabling the uptake of biologically active proteins/enzymes needed in cases of protein/enzyme deficiency, concentrating on mitochondrial diseases and on the import of enzymes or peptides in order to destroy pathogenic cells, focusing on cancer cells.

## 1. Introduction

Inherited metabolic disorders, caused by genetic aberrations most commonly inherited from both parents, affect the body’s metabolism and alters its ability to properly utilize proteins, fats and carbohydrates. People with inherited metabolic disorders have a defective gene that results in an enzyme/protein deficiency. There are hundreds of different genetic metabolic diseases, and their symptoms and prognoses, as well as possible available treatments, vary widely [1].

Treatments available for inherited metabolic disorders are very limited today and are based on attempts to: (1) reduce intake of any food or drug that cannot be metabolized correctly; (2) replace the enzyme/protein to restore metabolism as close to normal as possible; and (3) remove toxic products of metabolism accumulating due to the metabolic disorder [2,3]. In this review, we will discuss the approach of enzyme/protein replacement therapy as a means to treat metabolic disorders, focusing on metabolic mitochondrial disorders.

In addition, the idea of enzyme/protein delivery for pathological disorders has expanded in recent years. This approach aims not only to replace a mutated enzyme/protein with a wild type, functional metabolic enzyme/protein, as in the case of metabolic disorders, but also to deliver an active enzyme/protein in order to destroy specific pathological cells involved in various human diseases. In this regard, we will briefly describe the use of protein therapy, focusing mainly on delivering proteins/peptides of the apoptotic machinery for the possible treatment of cancer [4]. However, the major challenge of this promising approach to either rescuing or to destroying cells’ activity is the delivery of large molecules such as enzymes/proteins into the desired cells and organs.

## 2. Cell-Penetrating Peptides (CPPs) for Cellular and Intracellular Delivery

The cell membrane, a lipid bilayer, acts as a protective, permeable filter allowing only small, non-charged molecules to diffuse through it at considerable rates, while hydrophilic macromolecules with a molecular weight above 0.5–1.0 KDa have poor membrane permeability [5]. However, many therapeutic approaches involve targets inside the cell. Therefore, the ability to deliver large molecules such as enzymes/proteins across the cells’ membrane and/or intracellular membranes, thus reaching sub-cellular organelles such as the mitochondria, still poses great challenges in research and medicine [6].

Indeed, due to these limitations, many promising approaches, mainly protein-based, were not developed in the past to their full therapeutic potential [7]. Thus, the necessity of finding effective means of promoting the delivery of therapeutic compounds across the cell’s membrane is urgent. Various synthetic protein delivery systems such as lipid- or polymer-based nanocarriers have been developed and tested over the years. These include liposomes, polymer nanoparticles, polymersomes (stable polymeric vesicles, prepared using amphiphilic block polymers of different molecular weights [8]), micelles (formed by spontaneous arrangement of amphiphilic block copolymers in aqueous solutions [9]), nanogels (hydrogels with a three-dimensional tunable porous structure and a particle size in the sub-micrometer range [10]), and inorganic nanoparticles (such as carbon nanotubes and silver/gold/magnetic/ZnO/CuO-based nanoparticles [11]). In addition, protein delivery systems contain cell-penetrating peptides (CPPs), also called protein-transduction domains (PTDs) as carriers [12]. This review will focus on CPPs for delivery of enzymes/proteins into cells. CPPs are relatively short peptides, 8 to 30 amino acids, that can promote the delivery of a variety of molecules into cells, including proteins, nucleic acids and synthetic drugs (reviewed in [13]).

The first CPP was discovered in the late 1980s when a number of research groups studying the human immunodeficiency virus (HIV) reported the PTD of the trans-activator of transcription protein, Tat [14,15,16]. TAT is an 11-amino acid (residues 47–57) arginine- and lysine-rich sequence of the Tat protein encoded by HIV-1 [14,15,16]. TAT-cargoes are rapidly and efficiently internalized into cultured cells, intact tissues, and live tissues, while retaining their biological activity [17,18] Most importantly, TAT and TAT-cargoes can cross the blood-brain barrier (BBB), so are extremely useful for delivering proteins into tissues and mainly into the brain. Over the last decades, CPPs have attracted a great deal of interest for their potential in both research and medicine. Many CPPs, composed of naturally derived sequences and with the ability to get into cells have been characterized. In addition, a large number of chimeric and synthetically designed peptides have been constructed, aiming at improving cellular uptake and enhancing cellular and sub-cellular specificity.

### 2.1. CPP Classification

CPPs may be classified according to a number of different criteria. One common classification relates to their hydrophilicity and hydrophobicity that determine their different interactions with biological membranes. The first group is composed of cationic CPPs, represented by the HIV-1 protein TAT [19]. These peptides have a high content of arginine, lysine and histidine residues. These cationic peptides interact with the negatively charged phosphates and sulphates on the cell membrane, leading to its internalization under physiological pH conditions. Several studies have demonstrated that it is necessary to have at least eight positive charges for efficient cellular internalization [17].

The second group of CPPs is comprised of membranotropic and amphipathic peptides, consisting of hydrophobic amino acids with a low net charge. Amphipathic CPPs are sub-divided into primary, secondary α-helical, β-sheet, and proline-rich amphipathic CPPs [20]. These peptides contain lipophilic and hydrophilic moieties that are involved in promoting the peptide translocation across the cell membrane [21]. Viral fusion peptides belong to this group of CPPs [22].

### 2.2. CPP-Cargo: Complex Formation and Internalization

CPPs must be connected to their cargoes to deliver various biological molecules, such as proteins/enzymes into cells. Two different approaches are used for linking CPPs and cargoes: non-covalent, and the more common covalent connection producing a CPP-protein/peptide fusion molecule. For example, amphipathic peptides, such as MPG and Pep-1, can form stable complexes with various cargoes without the need for additional crosslinking or chemical modifications [23,24]. MPG is a 27 amino acid peptide derived from the hydrophobic fusion domain of HIV gp41, responsible for cellular entry, and the hydrophilic nuclear import sequence of SV40 T antigen, which binds the cargo and also enables entry into the nucleus [25]. Pep-1 is derived from MPG [26]. Pep-1 has been mainly used to deliver small peptides and proteins into cells, while MPG has been shown to efficiently deliver small interfering RNA (siRNA) into cell lines [27,28]. The approach of linking a CPP and its cargo by a covalent bond has been broadly utilized, demonstrating promising results with various CPPs [23,29,30], as will be discussed in the present review.

For CPPs’ internalization across biological membranes, several models have been proposed; however, the exact mechanism remains yet to be deciphered. Several lines of evidence support both direct translocation through the cell membrane as an energy-independent mechanism, as well as endocytosis, which is energy dependent, as being involved in the cellular uptake of various CPPs and CPP-cargo molecules [31,32,33]. As a process which requires no energy, direct translocation is regarded as a single-step process, including mechanisms involving the formation of inverted micelles [34], pores [35], and the “carpet” model, in which CPP-cargoes are translocated into the cell by charge interaction with cell membranes covering its surface in a carpet-like manner. At local high CPP-cargo concentrations, cell fluidity is increased leading to cell membrane disruption, and CPPs or CPP-cargoes are transported into the cell [36,37].

Endocytosis, in which macromolecules are transported into cells in vesicles or vacuoles, involves two distinct steps: endocytic uptake followed by endosomal escape. Both caveolae and clathrin-independent or -dependent pathways have been documented [38,39,40], as well as micropinocytosis mechanisms [41,42,43]. It is also accepted that other factors including cell type, nature of CPP, nature of cargo, and amounts of CPP and cargo, influence efficiency of both the internalization process and the endosomal escape [31,32,33].

**Figure 1:**
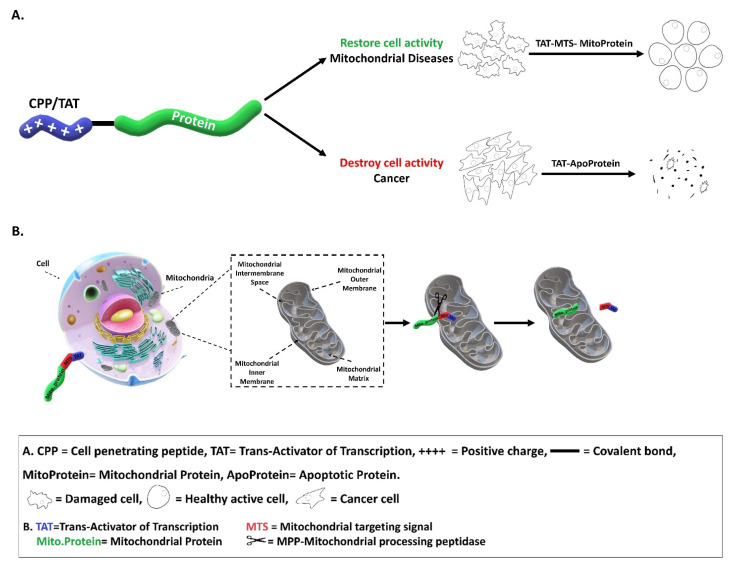
Therapeutic uses of CPPs (mainly TAT) in promoting the uptake of the biologically active proteins/enzymes for restoring or destroying cell activity for possible treatment of mitochondrial diseases or cancer, respectively (**A**). The principal of using TAT-MTS-MitoProtein fusion proteins for mitochondrial diseases (**B**).

The ability of CPPs to import various large molecules into cells and organs offers an opportunity to deliver active enzymes/proteins for therapeutic purposes. Here we focus on the potential therapeutic uses of CPPs (mainly TAT) as a delivery system of proteins/enzymes for the possible restoration of metabolic disorders, concentrating on mitochondrial diseases. We also describe the import of enzymes or peptides in order to destroy pathogenic cells, concentrating on cancer cells (summarized in Figure 1A). In addition, we will discuss the obstacles and limitations that must be overcome before applying these CPP/TAT-protein molecules for human use in medicine.

## 3. TAT-Fusion Protein Delivery for Enzyme/Protein Replacement Therapy (E/PRT)

The ability to deliver an active enzyme into cells has raised the possibility of developing many enzyme replacement therapies (ERT) using the TAT-based delivery system. ERT is a therapeutic approach to treat metabolic diseases whereby the deficient enzyme is produced, mainly as a recombinant protein, then purified and given to patients on a regular basis. At present, ERT is a successful treatment mainly for metabolic lysosomal storage diseases [44]. However, the inability of the delivered enzymes to cross the BBB severely limits this approach from being applied in the treatment of metabolic disorders involving the CNS [44]. Thus, the ability of CPPs, such as TAT, to deliver active enzymes across the cellular membrane and especially across the BBB, makes this delivery system highly promising for the development of therapies, in particular for mitochondrial diseases.

### 3.1. Mitochondrial Diseases and Existing Treatment Approaches

Mitochondrial diseases are a group of rare disorders affecting around 1 in 4300 live births and causing progressive, incurable defects that often result in premature death [45]. They have emerged as a major cause of inherited human diseases. These disorders are characterized by high genetic, biochemical, and clinical complexity that are caused mainly from dysfunction of the oxidative phosphorylation (OXPHOS) pathway, the major source for cellular energy production [46]. Impairment is caused by mutations in genes encoding (mainly by the nucleus) for proteins involved in the mitochondrial respiratory chain pathway, such as subunits of mitochondrial respiratory chain complexes, assembly factors, or by mutations in genes involved in other mitochondrial functions, including fission and fusion pathways, mitochondrial DNA (mtDNA) maintenance, iron/sulfur metabolism, and heme biosynthesis [47].

There are approximately 1500 predicted mitochondrial genes, which if mutated could lead to mitochondrial dysfunction and diseases [48]; more than 300 have been connected to mitochondrial disorders [49,50]. Advanced diagnostic technologies, such as next-generation sequencing, are leading to a rapid increase in identifications of disease-related genes. However, despite significant advances in understanding the pathophysiology underlying these diseases, the extremely heterogeneous phenotypes and symptoms of mitochondrial disorders [46] has greatly restricted the development of effective treatments. Currently, no approved cure exists for most mitochondrial disorders, and existing treatments are focused only on supportive management of the disease. Specific treatment of mitochondrial disorders is based on various approaches, such as increasing mitochondrial biogenesis, enhancing respiratory chain function, energy buffering, scavenging toxic compounds, vascular effects, or alteration of mitochondrial dynamics [51]. Treatment options include, among others, the use of various pharmacological cofactors and supplements, as briefly described here.

Coenzyme Q10, a component of the respiratory chain, is utilized to enhance the chain’s function. It is effective in cases of Q10 deficiency when given orally, demonstrating neuromuscular improvement, but without a clear neurological benefit, due to its inability to cross the BBB. However, Idebenone, a CoQ10 analogue that can cross the BBB, had not demonstrated significant efficacy in humans. In patients with mitochondrial encephalopathy, lactic acidosis, and stroke-like episodes (MELAS) or a history of stroke-like episodes, L-arginine is the treatment choice. It acts as a vasoactive agent via the nitric-oxide pathway [52]. Riboflavin (B2) has some evidence supporting its use in the treatment of complex I deficiency. L-carnitine participates in fatty acid transport into the mitochondria and is used to enhance fatty acid oxidation or in secondary carnitine deficiency [52]. Other supplements, such as thiamine and creatine, are given as chronic treatments; however, they have limited clinical benefit. Thiamine (B1), a cofactor of the pyruvate dehydrogenase complex (PDHC), can decrease lactate accumulation, while increasing acetyl-CoA production. In cases of myopathy or cardiomyopathy, patients are administrated creatine phosphate in order to increase ATP storage through the creatine phosphatase system, mainly in skeletal muscles.

Other medications include cysteine to increase the availability of the antioxidant glutathione peroxidase, nuclear respiratory factors, and peroxisomal proliferator activator receptors (PPARα,β,γ) and their agonists to enhance transcription of OXPHOS genes. EPI-743, a parabenzoquinone analog, exerts its antioxidant effects via repletion of reduced intracellular glutathione, and is a new therapeutic compound with evidence of reversal of disease [51]. There are some other compounds currently in clinical trials, including deoxycytidine monophosphate (dCMP) and deoxy thymidine monophosphate (dTMP), for treating thymidine kinase 2 (PK2) deficiency, a disease characterized by instability and depletion of mDNA [51].

Symptomatic treatment of mitochondrial disorders also includes organ-specific management, such as dialysis for renal dysfunction, ventilatory support, or surgical procedures [53]. Rarely, transplantation of organs such as the liver is the approach for treating severely affected patients [54]. However, despite the many approaches tested and several clinical trials that are being conducted, there is no specific treatment for mitochondrial disorders and their management is mainly supportive [51].

It should be emphasized that ‘repair’ of mitochondrial diseases is more complex than just successful replacement of a cytosolic enzyme or protein. Treatment approaches must take into consideration the requirement that the enzyme/protein be able to cross multiple mitochondrial membranes, as well as the fact that many enzymes in mitochondria are components of huge enzymatic complexes that need to be processed correctly in order to integrate and function properly. Additionally, many of the mitochondrial gene defects cause severe neurological symptoms and, as mentioned above, delivery of therapeutics across the BBB is difficult and still challenging.

Other approaches to treat mitochondrial diseases include E/PRT, shift of heteroplasmy rate, and stem cell and gene therapy; all these approaches are still experimental. E/PRT, however, is rapidly developing and is now in its very first Phase 1 clinical trials for a mitochondrial disease (see below for the mitochondrial frataxin protein).

### 3.2. Developing TAT-MTS-MitoProtein Molecules for Mitochondrial Diseases

In 2008, our lab [18] was the first to successfully use the TAT delivery system fused with the human mitochondrial lipoamide dehydrogenase (LAD) enzyme to deliver a mitochondrial enzyme into mitochondria, in the form of a TAT-protein (TAT-LAD) fusion protein. LAD, a nuclear-encoded mitochondrial protein, is one of the subunits of the α-ketoacid dehydrogenase complex in the mitochondrial matrix. In the case of LAD deficiency, the delivered, replacing enzyme needs to get into the cells and reach the mitochondria, be processed there and incorporated into three multi-component enzymatic complexes. We included the natural precursor sequence of human LAD consisting of the N-terminus 35 amino acid mitochondrial targeting sequence (MTS) to facilitate the processing of the TAT-LAD fusion protein upon its entry into the mitochondria, thus allowing the incorporation of the delivered LAD into the α-ketoacid dehydrogenase complexes. We demonstrated that the TAT-LAD fusion protein enters patients’ cells rapidly and efficiently, reaching the mitochondria. Inside the mitochondria, the TAT-LAD fusion protein is processed and restores LAD activity there. Most importantly, we showed that the TAT-LAD fusion protein was able to restore the activity of the whole PDHC mitochondrial complex, within treated patients’ cells, almost back to normal levels [18].

In addition, we showed that it was necessary to include the MTS sequence for maximal restoration of LAD enzymatic function. Deleting the MTS restored a significantly smaller amount of LAD activity within the mitochondria [18]. This is an important point to consider as TAT can move both ways across membranes and thus pull the replaced enzyme/protein out of the mitochondria. When the MTS is included, the matrix processing peptidases recognize the sequence and clip it off, thus leaving the replaced enzyme/protein (in this case the mature LAD) in the matrix in the same form as the natural endogenous human enzyme. The clipped-off TAT peptide can transduce out of the mitochondrion or can be degraded. Repeated dosing should therefore result in accumulating amounts of delivered enzyme/protein in the mitochondria over time. Figure 1B summarizes this promising approach of using TAT-MTS-MitoProtein fusion proteins for the treatment of mitochondrial disorders. Next, we used the TAT-LAD fusion protein to rescue LAD deficiency in mice [55]. TAT-LAD fusion proteins were delivered and could be detected within multiple tissues, mainly within highly energy demanding organs, including the liver, heart, and, most importantly, the brain (across the BBB). TAT-LAD fusion protein increased LAD enzymatic activity in those tissues. This was true not only for the LAD enzyme itself; the activity of whole PDHC in the mice organs was restored.

Very similarly, Foltopoulou et al. [56] demonstrated that when TAT was fused with Sco2, a nuclear-encoded and hydrophobic complex IV assembly protein involved in mitochondrial copper transfer, the fusion protein entered the mitochondria and partially recovered complex IV activity in K562 cells and patients’ fibroblasts. In a recent study [57], the authors radiolabeled the TAT-L-Sco2 fusion protein with technetium-99m to assess its in vivo bio-distribution. The distribution pattern of [99mTc]Tc-TAT-L-Sco2 in mice demonstrated fast blood clearance, and significant hepatobiliary and renal clearance. In addition, the TAT-L-Sco2 fusion protein was detected in the isolated mitochondria of a number mouse tissues, including the heart, muscle and brain [57].

Perry et al. [58] utilized the same approach and fused TAT with the *Saccharomyces cerevisiae* mitochondrial NADH-quinone internal oxidoreductase (Ndi1), and the fusion protein, TAT–Ndi1, entered cardiomyocytes and localized to mitochondrial membranes both in vitro and in vivo. When TAT–Ndi1 fusion protein was administrated into perfused rat hearts, it significantly ameliorated reperfusion injury [58,59].

Later, our lab [60] applied the TAT-cargo system to deliver the NADH dehydrogenase (ubiquinone) complex I assembly factor 4 (NDUFAF4; or C6ORF66) into patient cells. The TAT-C6ORF66 fusion protein, added to cultured patients’ cells, localized within the mitochondria, where it led to the proper assembly and most importantly, restored the function of complex I. Similarly, Lin et al. [61] conjugated TAT with NADH dehydrogenase (ubiquinone) Fe–S protein 8 (NDUFS8), the first complex I subunit linked to Leigh syndrome, and the resulting TAT-NDUFS8 or NDUFS8-TAT fusion proteins were successfully delivered into mitochondria. Mitochondrial delivery was independent of mitochondrial membrane potential. Moreover, treating with TAT-NDUFS8 could fully restore the assembly and partly recover function of complex I in NDUFS8-knockdown cells. These results further confirmed the notion that TAT-mediated ERT is a very promising treatment approach to rescue mitochondrial protein deficiency.

In our lab, we tested the TAT-E/PRT treatment approach also for the possible rescue of methylmalonic aciduria (MMA), a disorder of organic acid metabolism, defective in the mitochondrial enzyme, methylmalonyl-CoA mutase (MCM; see below). Today, the main treatments for MMA patients are dietary restriction of propiogenic amino acids and carnitine supplementation. The very risky and complicated liver or combined liver/kidney transplantation treatment is for those with the most severe clinical manifestations.

It should be pointed out that in all published research on TAT-MitoProtein fusion proteins, the native MTS of the mitochondrial enzyme/protein is used to construct the fusion protein. However, in our lab, we also used heterologous MTSs (hMTSs) of human, nuclear-encoded mitochondrial proteins to target a human mitochondrial protein, instead of the native MTS. The hMTSs of three enzymes, citrate synthase (CS), LAD and C6ORF66 (ORF), which have classic MTS sequences and are known to be removed by one-step processing, were chosen to deliver MCM into mitochondria, in the form of TATMTSx-MCM fusion proteins (MTSx standing for the hMTS). This was performed for both the MCM protein [62] and frataxin protein ([63]; see below). In the case of the MCM protein, fusion proteins constructed with a heterologous MTS sequence reached the mitochondria and successfully underwent processing. Treatment of MMA patient fibroblasts with these TAT-MTSx-MCM fusion proteins restored mitochondrial activity including ATP production, mitochondrial membrane potential and oxygen consumption. Treatment with the fusion proteins enhanced also cell viability of patients’ cells and most importantly reduced MMA levels. Treatment also enhanced albumin and urea secretion in a CRISPR/Cas9-engineered HepG2 MCM^(−/−)^ liver cell line, generated in our lab. In addition, results of this study emphasized the importance of proper mitochondrial function in MMA.

Another mitochondrial disorder that was proposed to be suitable for the TATMitoProtein fusion protein treatment approach is propionic acidemia, caused by a deficiency of the mitochondrial enzyme propionyl coenzyme A carboxylase (PCC). The TAT-PCC fusion protein was indeed delivered into mitochondria of patient fibroblasts [64]. Moreover, a single-dose intraperitoneal injection into PCC-deficient mice decreased the propionylcarnitine/acetylcarnitine (C3/C2) ratio to about the normal level. These results showed again that CPP/TAT can deliver active enzymes into mitochondria both in vitro and in vivo [64].

However, the most advanced studies for P/ERT as a possible treatment approach for mitochondrial diseases is the use of the TAT delivery system for the mitochondrial frataxin (FXN) protein, involved in Friedreich’s ataxia (FRDA). This is a progressive, neurodegenerative disorder and the most common autosomal recessive ataxia worldwide, affecting approximately 1 in 50,000 people. FRDA is caused by biallelic intronic GAA repeat expansion in the frataxin gene on chromosome 9, ranging from 66 to approximately 1300 GAA repeats [65]. The expanded GAA repeats lead to transcriptional silencing of frataxin, causing a deficiency of FXN protein [66]. The FXN protein is a mitochondrial protein that plays an important role in iron homeostasis. The deficiency of FXN protein results in mitochondrial dysfunction, subsequently leading to decreased production of ATP, impaired iron-sulfur cluster assembly, abnormal iron accumulation, generation of reactive oxygen species, increased oxidative stress, and ultimate cell damage. Genetic testing is available for FRDA diagnosis confirmation. FRDA patients typically experience symptoms before age 25, usually in their early teenage years. Symptoms include gait and limb ataxia, dysarthria, areflexia, vibratory and positional dysfunction, and muscle weakness. Non-neurological symptoms include diabetes, cardiac hypertrophy, scoliosis, and others [66,67,68,69]. FRDA patients typically become wheelchair-bound approximately ten years after initial symptoms, and cardiomyopathy is usually the primary cause of premature death [70,71,72,73]. While FRDA patients have a severe reduction in FXN, carriers of one allele of the mutated gene, with approximately 50% of FXN levels, remain asymptomatic [69]. Therefore, increasing FXN levels to that of carriers remains an attractive therapeutic strategy. Much progress was achieved in understanding the genetic and molecular basis of the disorder; however, there is no cure for FRDA, nor there is an FDA-approved treatment. One possible promising approach for treatment is the use of a TAT-MTS-MitoProtein fusion protein.

FXN protein has been fused to TAT-protein, along with its native MTS, producing the TAT-FXN fusion protein [73]. Membrane processing proteases (MPPs) in the mitochondria remove the targeting sequence and the TAT peptide, leaving FXN in its native form ([73]; and Figure 1). In this way, FXN is delivered into the mitochondrial matrix as a protein, to elevate the low FXN levels found in FRDA patients. TAT-FXN augmented aconitase activity in an FXN-KO mouse heart, and improved cardiac function in this animal model [73]. Intraperitoneal TAT-FXN injections also rescued a fatal conditional knockout mouse model that had been stripped of neuronal FXN [74]. The same TAT-FXN fusion protein has also been shown by Kim et al. [75] to protect dopaminergic neurons against 1-methyl-4-phenyl-1,2,3,6-tetra-hydropyridine-induced toxicity in a mouse model of Parkinson’s disease.

As described above, MTSs are responsible for trafficking nuclear-encoded proteins into mitochondria. Once entering the mitochondria, the MTS is recognized and cleaved off. Some MTSs are unusually long and undergo two-step processing, as in the case of the human FXN protein (80 aa). Therefore, FXN protein was chosen by our laboratory [63] to examine whether nuclear-encoded mitochondrial proteins can efficiently be targeted via an hMTS and deliver a functional protein into mitochondria. We used the same hMTSs, CS, LAD and ORF as described for the MCM mitochondrial enzyme, to produce the TAT-MTSx-FXN fusion proteins. We demonstrated that using hMTSs for delivering FXN results in the production of 4–5-fold larger amounts of the fusion proteins, at 4–5-fold higher concentrations, thus most likely suggesting the production of TAT-MCM fusion proteins with enhanced stability and ease to produce. Moreover, hMTSs delivered a functional FXN protein into the mitochondria even more efficiently than the native MTSfxn, as evidenced by the rescue of FA patients’ cells from oxidative stress [63]. One fusion protein containing the MTScs increased aconitase activity within patients’ cells by 400-fold, highlighting the fact that when designing TAT-MTSMitoProtein fusion proteins, one should consider using mitochondrial hMTS, which can enhance mitochondrial delivery and thus increase efficacy of TAT-MTS-MitoProtein fusion proteins in the treatment of mitochondrial disorders.

Britti et al. [76] studied the ability of our TAT-MTScs-FXN fusion protein to improve neurodegenerative markers on frataxin-depleted dorsal root ganglia neurons, as well as whether treatment with TAT-MTScs-FXN fusion protein improved the lifespan of FRDA mice. Indeed, dorsal root ganglia neurons treated with TAT-MTScs-FXN fusion protein had significantly improved cellular survival, while FRDA mouse models had increased lifespans [76].

These promising results of TAT-FXN fusion protein in animal models indicate that it may be an effective modality to treat mitochondrial protein defects in life-threatening conditions such as FRDA. Indeed, the first Phase 1 (NCT04176991) clinical trial to evaluate the safety and efficacy of TAT-FXN fusion protein in the treatment of FRDA is currently underway [77].

## 4. Delivery of Proteins/Enzymes via CPPs for Cancer Treatment

Many newly developed treatments for cancers rely on protein therapy and utilize CPPs to deliver active enzymes/proteins in order to destroy and eliminate cancer cells. Suppression of apoptosis is central to the development of cancer and is associated with resistance to modern treatments. Therefore, molecules and pathways of apoptotic processes are attractive targets for the development of anticancer therapeutics. Moreover, induction of apoptosis in tumor cells, specifically within the tumor microenvironment, is required to kill them efficiently and to enhance the effects of more conventional treatments. Apoptosis is executed by intracellular proteins; therefore, molecular approaches, such as the use of CPPs, must be incorporated to deliver the treatment into the tumor cells. Here we focus on the use of CPP/TAT-ApoProtein fusion proteins for the possible treatment of cancer (see Figure 1A above).

### 4.1. CPP/TAT-ApoProtein Fusion Proteins for Cancer Treatment

CPPs, mainly the TAT peptide, have been fused to various proteins of the cellular apoptotic machinery, both to those of the death-receptor mediated apoptotic pathway and to those of the intrinsic mitochondrial apoptotic pathway. One such protein is BID, a BH3-only pro-death Bcl-2 family molecule that can interact with the pro-death molecules Bax or Bak, enabling it to either inactivate the anti-death molecules and/or directly activate a pro-death molecule. BID, as a caspase-truncated product (tBID), triggers the mitochondrial apoptotic program following death-receptor activation, thus linking the two major apoptotic pathways. Recombinant BID fused with TAT peptide, the TAT-BID fusion protein, internalized into a number of cancer cells in amounts depending on time, dose and the cell line. TAT-BID fusion protein sensitized PC3 cells to anticancer agents acting through death receptors (TRAIL) or DNA damage [78].

Another example is TAT-Bim, in which TAT was fused to a biological effector domain, the BH3 domain from the pro-apoptotic Bcl-2 family member Bim [79]. TAT-Bim fusion protein internalized into cancer cells within a short incubation time. Treatment with TAT-Bim fusion protein resulted in apoptosis in a dose-dependent manner and sublethal irradiation augmented the effects of TAT-Bim induced apoptosis. Treatment with TAT-Bim fusion protein also significantly slowed tumor growth in murine models of pancreatic cancer and melanoma [79]. In a recent publication, the CPP and Bim parts of the fusion peptide were systematically shortened, and the pro-apoptotic activities of the shortened peptides were examined [80]. The authors obtained TatBim-N1C2 and R8Bim-N1C2 as minimized peptides with efficient apoptotic activity that may be good candidates for cancer therapy [80].

Similarly, the human Fas-associated death domain (FADD) protein was chemically conjugated to the TAT peptide for delivery into cancer cells [81]. The authors investigated the potential of the TAT conjugated FADD (TAT~FADD) to interfere with apoptosis and NF-κB signaling within cancer cells. The FADD protein, together with pro-apoptotic proteins, such as procaspase-8/10, is involved in the formation of the multimeric death-inducing signaling complex (DISC), an essential step in the dead-receptor mediated apoptotic pathway. The authors demonstrated that the TAT~FADD conjugate efficiently entered colon carcinoma HCT116 cells and formed a DISC, followed by increase of apoptotic signaling in the cancer cells. Moreover, TAT~FADD targeted also NF-κB signaling, suppressing the expression of a number of anti-apoptotic genes such as Bcl2, cIAPs, RIP1, and cFLIPL in those colon cancer cells. [81].

Second mitochondria-derived activator of caspase (Smac) is another key pro-apoptotic pathway, activated with a Smac mimetic peptide. Priwitaningrum and his team [82] developed a nano-system to deliver a Smac peptide to tumors. They first synthesized a chimeric peptide that consists of the 8-mer Smac peptide and a 14-mer cell CPP and then encapsulated the Smac-CPP into polymeric nanoparticles (Smac-CPP-NPs). Smac-CPP-NPs rapidly internalized into 4T1 mammary tumor cells and subsequently released Smac-CPP into the cells, leading to induction of apoptosis in the tumor cells. In addition, the combination of Smac-CPP-NPs with doxorubicin (dox) significantly improved the apoptotic effect compared to that of SMAC-CPP-NPs alone and that of control empty nanoparticles and dox. Combination treatment with Smac-CPP-NPs and dox reduced the tumor growth by 85%. This study demonstrated that the intracellular delivery of Smac-mimetic peptide by a CPP, using a nanoparticle system, could be a promising strategy to reduce tumor growth and to potentiate the therapeutic efficacy of chemotherapy in vivo [82].

### 4.2. CPPs for Delivering Vaccines for Cancer Therapy

In the past decade, many research groups have used CPPs for the development of vaccine delivery systems [83]. The aim is to deliver antigenic peptides into antigen-presenting cells in order to process and present them properly, thus leading to induction of an immune response. Pouniotis et al. used the CPP, Penetratin, and linked it to cytotoxic T lymphocyte epitopes derived from ovalbumin (OVA) or mucin-1 tumor-associated antigens. These complexes were able to induce stimulation of CD4+ and CD8+ T cells in vitro. In addition, the complexes evoked a T cell response in vivo, leading to the secretion of cytokines and consequently to inhibition of B16.OVA melanoma cell growth. Moreover, pre-immunization with Penetratin-OVA protects mice from a following tumor challenge [84,85]. Brooks et al. demonstrated the therapeutic potential of a CPP linked to mucin-1 and T cell epitopes. In a tumor vaccination model, these CPP-peptides induced a number of immune responses and delayed mucin-1 tumors in mice [86]. Searching for an efficient CPP for delivery of cancer-related antigens into cancer cells, Shahbazi and Bolhassani [87] performed a comparison of six CPPs for the delivery of the HPV16 E7 antigen. E7 is an oncoprotein constitutively expressed by HPV-infected cells, and therefore represents a therapeutic vaccine target. They demonstrated that a number of CPPs could efficiently deliver the E7 antigen into tumor cells and that immunization of mice induced long-term protection against tumor challenge, especially in the group treated with p28-E7; p28 representing the CPP component of the molecule.

It should be pointed out that p28 itself has anti-tumoral activity. The penetrating properties of p28 are due to a domain derived from the protein azurin (amino acids 50 to 67), called p18 [88]. The full-length peptide prevented p53 ubiquitination in cancer cells, and induced a post-translational increase of p53 [89]. The peptide was able to inhibit angiogenesis and cancer cell growth [89,90]. In addition, p28 was found to preferentially enter tumor cells [91]. Noei and his team designed a novel therapeutic molecule, based on the fusion of p28 to Apoptin (p28-Apoptin) [92]. Apoptin accumulates in the nucleus of tumoral cells, where it induces apoptosis [93]. The authors showed that p28-Apoptin fusion protein caused a selective cytotoxicity of breast cancer cells [92].

### 4.3. CPPs for the Modulation of Protein-Protein Interactions (PPIs) in Cancer Therapy

Protein–protein interactions (PPIs) are involved in almost every cellular process and regulate numerous signaling pathways. Thus, targeting and destroying PPIs in cancer may represent an efficient therapeutic approach. Tian et al.’s work focused on disrupting the SET-PP2A interaction with cancer cells [94]. SET is an oncoprotein that inhibits the activity of PP2A, a known tumor suppressor [95]. They constructed two peptides composed of the CPP, Mut3DPT, linked to either SET’s or PP2A’s interaction sites, Mut3DPT-SET and Mut3DPT-PP2A, respectively. The authors demonstrated that these CPP-peptides destroy SET-PP2A interaction and indeed exhibit an anti-tumor effect both in vitro and in vivo. Similarly, Ras/Raf activation is involved in the development of lymphoid cancers. Therefore, the same research team constructed also a Ras/Raf interfering peptide linked to the CPP Mut3DPT [96]. Their CPP-peptide induced tumor cell death in chronic lymphocytic leukemia (CLL) cell lines and primary cells. Treatment with the CPP-peptide also prolonged survival in CLL mice models [96].

Other protein–protein interactions were identified in recent years that could be potential targets for cancer therapeutics [97]. One such example was reported by Poyet and his team, showing that RT53, a cell-permeable peptide derived from the survival protein AAC-11, selectively killed cancer cells in vitro and prevented tumor growth in melanoma mouse models in vivo [98,99]. RT53 inhibited the AAC-11’s anti-apoptotic properties, via disruption of protein-protein interactions between AAC-11 and cellular protein partners such as Acinus [98,99]. B16F10 mouse cells treated with RT53 were able to mediate protective effects in a tumor vaccination model [98,99]. In a recent study [100], the authors demonstrated that RT53 had a direct anti-leukemic effect, both in vitro in cancer cell lines, and in vivo, in an acute promyelocytic leukemia (APL) mouse model. A vaccine consisting of RT53-exposed leukemic blast cells was highly effective at preventing leukemia development both prophylactically and therapeutically via the induction of CD4 + T cell-dependent long-term response.

## 5. Obstacles/Limitations of Utilizing Recombinant CPP-Enzymes/Proteins for Human Therapy

Fusion or conjugation of therapeutic proteins/enzymes/peptides to CPPs shows great promise for developing novel therapies, as described in this review; however, there are still many major challenges to overcome, including the following:

1. Cell/tissue-specific targeting of CPP-protein modalities. One major challenge in using enzymes/proteins for therapy is delivering them to the specific site of action, the target tissue/cells. This is less important in the case of mitochondrial disorders, since the replaced protein/enzyme should reach all cells and organs, as demonstrated in the present review. In the case of cancer treatment, specificity is a major requirement in the development of CPP-protein molecules as anticancer therapeutics. One way to obtain specificity towards cancer cells is to develop tumor-specific CPPs using, for example, phage-displayed peptide libraries ([101]; see also the RGD peptide below) or conjugating a third molecule that bestows specificity to the CPP-cargo molecule [102]. Another strategy to improve specificity to tumor cells is using monoclonal antibodies (mAb). For example, Shin and his research team designed a complex system comprised of two conjugates, a conjugate of heparin and an anti-carcinoembryonic antigen (anti-CEA) monoclonal antibody in combination with a TAT-gelonin fusion protein, gelonin being an inhibitor of protein synthesis [103,104]. Another approach is to use a system in which the CPP’s activity is masked with an anionic peptide by a cleavable linker. Once in the tumor tissue, degradation of the linker activates the CPP [104]. Tsien and his team used a cleavable linker that is recognized by the matrix metalloproteinase-2 (MMP-2), known to be upregulated in most solid tumors [105]. Another approach relies on cellular mechanisms specific to tumor cells. One such example is hypoxia, a phenomenon common to most cancer cells. Hypoxia-inducible factors (HIFs) are the main effectors of the cell’s response to hypoxia; stabilization and up-regulation of HIFs proteins contribute to cancer development and progression. Overexpression of ERK-targeted domain (ETD) variants causes HIF-1 inactivation [106]. Therefore, Karagiota et al. constructed TAT-EDT peptides to target cancer cells under hypoxia in hepatoma-carcinoma cell models, and demonstrated a specific cytotoxic effect against these tumor cells [107]. In recent years, some further tumor-specific peptides were identified, such as the tripeptide motif Arg-Gly-Asp (RGD), which is able to recognize specific integrins expressed on cancer cells [108,109]. Zhou et al. characterized a new CPP called MT23 with mouse melanoma cell specificity, and demonstrated that a MT23-Apoptin fusion protein they constructed can significantly inhibit tumor growth and induce apoptosis in B16 melanoma tumor model in mice [101].
2. Targeting to specific intracellular compartments/organelles. The use of TAT–MTS-MitoProtein fusion proteins to treat mitochondrial diseases is an excellent approach to resolve the problem of specific sub-cellular targeting, as described in this review. The main concept is to include the MTS (a natural or a heterologous one) in the CPP/TAT-MitoProtein fusion protein that is recognized by the endogenous mitochondrial machinery and is clipped-off once entering the mitochondria, hence delivering a natural enzyme/protein into the mitochondria. Based on the same idea, using a nuclear localization signal (NLS) together with a CPP can achieve both cell and nuclear targeting. Wang et al. [110] used the NLS from simian virus 40 large-T antigen with octa-arginine to promote DNA delivery into the nucleus. The NLS-octa-arginine molecules effectively delivered luciferase DNA to various cell lines [111]. A cyclic new CPP, cyc 3 (derived from an antimicrobial peptide), was found to localize to the nucleus [111]. Upon incubation with cells, the peptide demonstrated a high level of nuclear localization, indicating its potential use for future therapeutic applications [112].
3. Stabilization of the CPP-protein/enzyme molecules. Stabilization of CPP/TAT-protein fusion proteins is necessary in order to delay of degradation of CPP-protein fusion proteins by enzymes circulating in the plasma. Replacing some amino acids of the CPPs can improve stability in vivo. For example, natural amino acids can be changed with unnatural amino acids, i.e., lysine with ornithine [113], and L-amino acids can be replaced by D-amino acids [114]. It is also possible to design protease-resistant CPPs using a shielding technique. Cationic CPPs can interact with negatively charged polymers, such as polyethylene glycol (PEG). Addition of PEG protects CPPs against degradation, enhancing both its metabolic stability in the plasma and a longer biological half-time [115]. However, it is important to find the delicate balance between enhanced stability to ensure the delivery of the CPP-protein molecules to the targeted cellular component and its sufficient clearance to avoid non-specific toxicity.

For example, it is possible to enhance cellular uptake by changing peptides into cyclic peptides [29,116,117], dendrimers [118], or changing their side chains [119,120,121]. Addition of tri-fluoro-methyl-quinoline moieties [122] or change of certain amino acids with histidine are also utilized to enable endosome escape of CPPs [123,124]. Oskolkov et al. designed stearylated TP10 analogs, named NickFects, with enhanced endosomal escape efficiency [125]. Overall, it is important to ensure that modifications of CPPs will not alter their solubility, immunogenicity, or toxicity.

4. Immune response to administration of CPPs and CPP-protein complexes. CPP sequences originate from non-human proteins/peptides and therefore are usually novel to the organism, thus possibly evoking an immune response [126]. Additionally, fusing of a CPP such as TAT with an enzyme or protein will most likely result in the generation of new epitopes that might also elicit an immune response [126]. Moreover, in the case of genetic metabolic disorders the replacement of the deficient enzyme or protein would have to be on a regular, chronic basis, causing additional problems regarding the immune response to the delivered CPP-protein modalities [6]. It is therefore of vast importance to evaluate the potential immunogenic effects of CPPs or CPP-enzyme/protein molecules.
5. Toxicity of CPPs and CPP-protein complexes. Numerous reports have demonstrated [6,127] that it is possible to safely inject/deliver CPP-enzyme/protein molecules into animal models as well as to humans (first Phase 1 clinical trial of TAT-FXN fusion protein [77]). Nevertheless, particularly careful tests should be performed to search for non-specific toxicity and safety aspects, when dealing with this new group of CPP-cargo therapeutic modalities.

## 6. Summary and Concluding Remarks

In the recent decades, many efforts have been devoted to finding novel protein/enzyme-based therapies for human diseases, focusing mainly on finding ways to deliver them into cells and organs. CPPs are commonly utilized for developing enzyme/protein-based modalities, based on their ability to deliver various large cargoes, including enzymes/proteins, into cells. However, there is still no FDA-approved CPP-protein drug, although CPP-protein molecules are currently in their first clinical trials, both for replacing the mitochondrial frataxin protein (TAT-FXN; [77]) and for destroying cells as in the case of p28 for the treatment of solid p53 tumor [91].

CPP-protein molecules are attracting, promising reagents to treat metabolic disorders; however, a number of issues should be addressed, in the next few years, before translating CPP-protein molecules into clinical use for humans. These include mainly the non-specific cellular uptake, the stability in vivo, and the route of administration. Good progress has been made to improve these limitations; however, much more research is needed before CPP-proteins can become commonly and widely approved drugs for the treatment of genetic metabolic diseases, whether for restoring a metabolic enzyme/protein deficiency, as in the case of treating incurable mitochondrial disorders, or for destroying pathogenic cells, as in the case of treating cancer.

## Data Availability

Not applicable.
